# Effect of current direction and muscle activation on motor cortex neuroplasticity induced by repetitive paired‐pulse transcranial magnetic stimulation

**DOI:** 10.1111/ejn.16099

**Published:** 2023-07-27

**Authors:** Ryoki Sasaki, Wei‐Yeh Liao, George M. Opie, John G. Semmler

**Affiliations:** ^1^ Discipline of Physiology University of Adelaide Adelaide Australia

**Keywords:** motor‐evoked potential, plasticity, primary motor cortex, repetitive transcranial magnetic stimulation

## Abstract

Repetitive paired‐pulse transcranial magnetic stimulation (TMS) at indirect (I)‐wave periodicity (iTMS) can increase plasticity in primary motor cortex (M1). Both TMS coil orientation and muscle activation can influence I‐wave activity, but it remains unclear how these factors influence M1 plasticity with iTMS. We therefore investigated the influence of TMS coil orientation and muscle activation on the response to iTMS. Thirty‐two young adults (24.2 ± 4.8 years) participated in three experiments. Each experiment included two sessions using a modified iTMS intervention with either a posterior–anterior orientation (PA) or anterior–posterior (AP) coil orientation over M1. Stimulation was applied in resting (Experiments 1 and 3) or active muscle (Experiments 2 and 3). Effects of iTMS on M1 excitability were assessed by recording motor evoked potentials (MEPs) and short‐interval intracortical facilitation (SICF) with PA and AP orientations in both resting (all experiments) and active (Experiment 2) muscle. For the resting intervention, MEPs were greater after AP iTMS (Experiment 1, *P* = .046), whereas SICF was comparable between interventions (all *P* > .10). For the active intervention, responses did not vary between PA and AP iTMS (Experiment 2, all *P* > .14), and muscle activation reduced the effect of AP iTMS during the intervention (Experiment 3, *P* = .002). Coil orientation influenced the MEP response after iTMS, and muscle activation reduced the response during iTMS. While this suggests that AP iTMS may be beneficial in producing a neuroplastic modulation of I‐wave circuits in resting muscle, further exploration of factors such as dosing is required.

## Graphical abstract

We examined the effect of repetitive paired‐pulse transcranial magnetic stimulation (TMS) at indirect (I) wave intervals (iTMS) on corticospinal excitability. We found that TMS coil orientation (posterior–anterior iTMS vs. anterior posterior iTMS) influenced the response to iTMS in resting muscle (Experiment 1) whereas muscle activation reduced the effectiveness of iTMS during the intervention (Experiments 2 and 3).

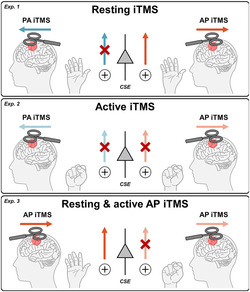

**Abbreviations**
AMT: active motor threshold
AP: anterior–posterior
CS: conditioning stimulus
EMD: estimated mean difference
EMG: surface electromyography
EMM: estimated marginal means
FDI: first dorsal interosseous
GLMM: generalised linear mixed model
iTMS: repetitive paired‐pulse transcranial magnetic stimulation
M1: primary motor cortex
MEP: motor‐evoked potential
MSO: maximum stimulator output
MVC: maximal voluntary contraction
PA: posterior–anterior
RMT: resting motor threshold
TMS: transcranial magnetic stimulation
TS: test stimulus

## 1 INTRODUCTION

Neural plasticity refers to the capacity of the brain to functionally and structurally modify itself (for review, see von Bernhardi et al., 2017), which is essential for learning and memory (for review, see Sweatt, 2016), and recovery from brain injury (for review, see Nudo, 2013). Transcranial magnetic stimulation (TMS) is a commonly used non‐invasive approach for investigating neural plasticity: This technique uses a strong magnetic pulse to activate areas of cortex via electromagnetic induction (Barker et al., 1985). When applied over primary motor cortex (M1), TMS generates a volley of activity in corticospinal neurons that summates at the spinal cord to produce a motor‐evoked potential (MEP) within target muscles, the amplitude of which provides an indirect measure of corticospinal excitability. However, applying trains of stimuli can produce changes in corticospinal excitability that are thought to reflect the induction of neuroplasticity in the brain (Hamada et al., 2007; Huang & Rothwell, 2004; Stefan et al., 2002).

The corticospinal descending volley generated by TMS involves several waves with complex origins that are not well understood. Despite this, it is recognised that the earliest wave reflects direct activation of the corticospinal neuron (and is therefore referred to as a D‐wave) and the following waves reflect indirect activation via recruitment of local intracortical circuits (di Lazzaro et al., 2001). These indirect (I) waves occur with a periodicity of ~1.5 ms and are numbered in order of appearance (I1, I2, I3, etc.). Importantly, TMS paradigms that induce neuroplasticity also result in specific alterations to I‐wave amplitude, providing evidence that the changes in excitability produced by these interventions are cortical in origin (di Lazzaro et al., 2008). In addition, more recent work has shown that specific I‐waves have unique functional roles (Hamada et al., 2014). Consequently, the targeted manipulation of different I‐wave circuits represents a potentially powerful approach to induce specific functional benefits.

Although existing plasticity paradigms can modulate I‐wave amplitude (for review, see di Lazzaro et al., 2012), this was generally not a feature of their design. In contrast, I‐wave periodicity repetitive TMS (iTMS) was developed specifically to target the I‐wave generating circuits (Thickbroom et al., 2006), although it is likely to also activate cortical circuits in addition to those involved in I‐wave generation (di Lazzaro et al., 2007). This paradigm involves the repeated application of stimulus pairs timed to coincide with I‐wave periodicity. Changing the timing of stimuli therefore means iTMS can be modified to target different elements of the descending volley, and preliminary work has shown this to be functionally beneficial (Long et al., 2017). However, we have recently shown that altering the timing of iTMS may have limited effects on which I‐wave circuits are targeted by the intervention (Sasaki et al., 2023). Investigating alternative approaches to target different I‐wave circuits is therefore important.

While altering the timing of stimulation is one approach to potentially influence I‐wave recruitment, an alternative approach is to instead change the direction of cortical current induced by TMS. For example, stimulation is conventionally applied to induce a posterior to anterior (PA) current (relative to the central sulcus), which preferentially recruits I1‐waves. In contrast, an anterior to posterior (AP) current instead results in preferential recruitment of later I‐waves (di Lazzaro et al., 2001). Furthermore, neuroplasticity induced by paired associative stimulation (PAS) has been shown to be more effective when AP TMS is applied during activation of the target muscle (Kujirai et al., 2006). Despite this, iTMS has only ever been applied in resting muscle with a PA current. Within the current study, we therefore aimed to investigate if changes to current direction and activity state can alter the I‐wave circuits targeted by iTMS. We expected that iTMS applied with a PA current would target early I‐waves, whereas iTMS with an AP current would target later I‐waves. However, we also expected that the response to these interventions would be influenced by muscle activation.

## 2 METHODS

### 2.1 Participants

Thirty‐two healthy young adults (12 men and 20 women; mean age ± SD = 24.2 ± 4.8 years; age range = 19–35 years) were recruited from the university and wider community to participate in the current study. Based on self‐reporting, 31 participants were right‐handed, and one was left‐handed. All participants were free of neurological and psychiatric disorders and were not taking any drugs that influence the central nervous system. Contraindications to TMS were assessed using the TMS adult safety screening (Rossi et al., 2009). A nominal reimbursement of $15 per hour was offered to compensate for time and cost of participation. Written‐informed consent was provided prior to inclusion, and the study was conducted in accordance with the *Declaration of Helsinki*. All experimental procedures were approved by the University of Adelaide Human Research Ethics Committee (approval number: H‐026‐2008).

### 2.2 Experimental arrangement

**FIGURE 1:**
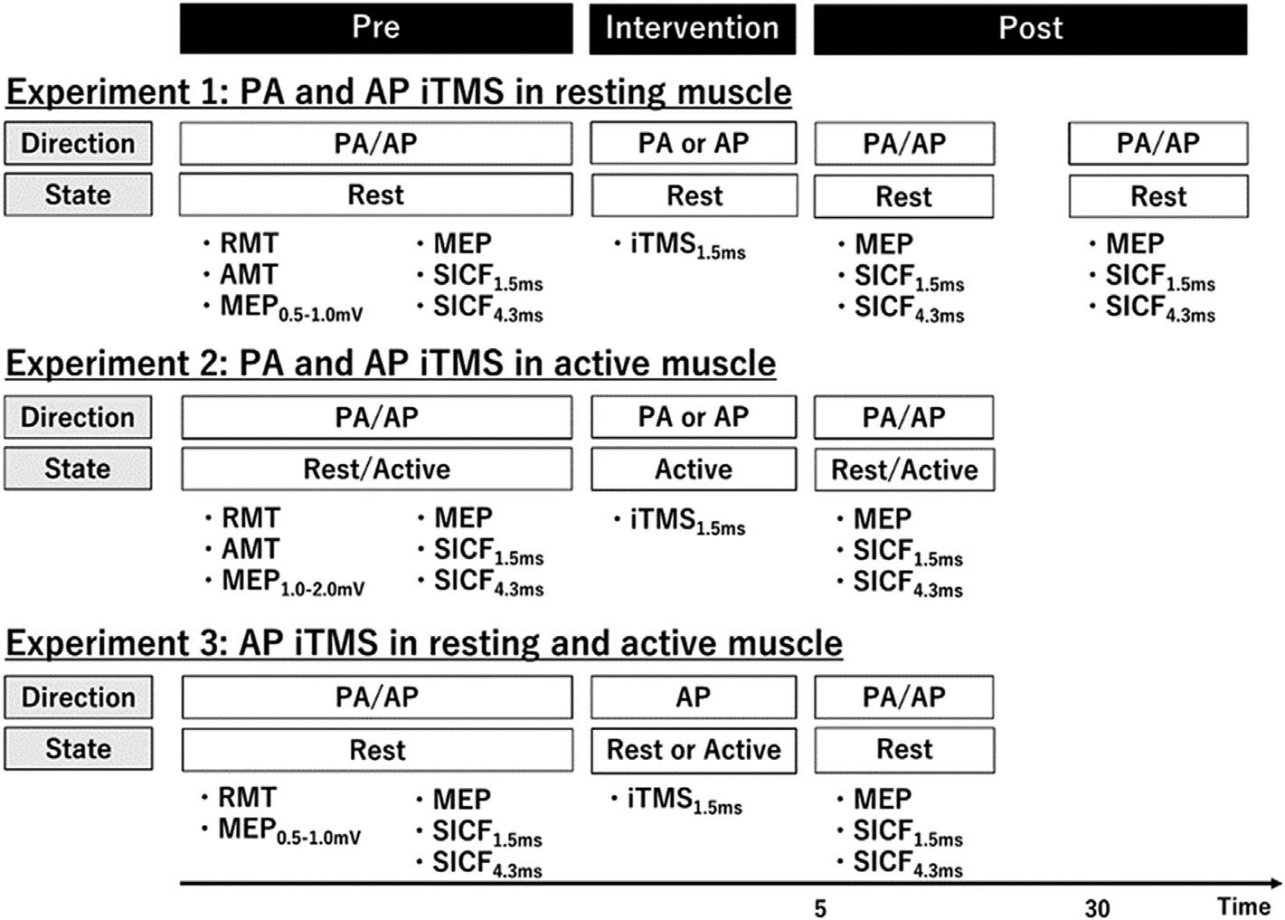
Experimental protocol. Each experiment involved two experimental sessions that were separated by at least 1 week. Experiment 1 applied 10 min iTMS (PA and AP orientations) at rest and recorded changes in MEPs/SICF with PA and AP orientations in resting muscle. Experiment 2 applied 5 min iTMS (PA and AP orientations) during weak muscle contraction and recorded changes in MEPs/SICF with a combination of coil orientations (PA and AP) and activation states (rest and active). Experiment 3 involved 5 min iTMS (resting and active muscle) with AP orientation and recorded changes in MEPs/SICF with PA and AP orientations at rest. Abbreviations: AMT, active motor threshold; AP, anterior–posterior; iTMS, I‐wave periodicity repetitive transcranial magnetic stimulation; MEP, motor‐evoked potential; PA, posterior–anterior; RMT, resting motor threshold; SICF, short‐interval intracortical facilitation.

Within each experiment, participants visited the laboratory for two sessions that were approximately 2 h long, held at the same time of day and were separated by a period of at least 1 week (Figure 1). As diurnal variations in cortisol may influence the neuroplastic response to TMS (Sale et al., 2007), experimental sessions were performed after 11:00 AM (around 11:00 AM or 2:00 PM). The order of iTMS sessions, measures in different coil orientations (PA and AP) and measures in different states of muscle activation (rest and active) were randomised within a participant. The project involved three separate experiments (see below), with 1 participant contributing data to all three experiments, 13 participants contributing data to two out of three experiments and 18 participants contributing data to one out of three experiments.

During each experimental session, participants were seated in a comfortable chair with their right hand pronated on an arm rest and were instructed to keep their eyes open. Surface electromyography (EMG) was recorded from the right first dorsal interosseous (FDI) muscle via disposable Ag/AgCl electrodes arranged in a belly–tendon montage on the skin above the muscle, with an additional Ag/AgCl electrode placed over the right ulnar styloid as an earth. EMG signals were sampled at 2 kHz using a CED1401 interface (Cambridge Electronic Design, Cambridge, United Kingdom), amplified (1,000×) and band‐pass filtered (20–1000 Hz) by a CED1902 signal conditioner (Cambridge Electronic Design, Cambridge, United Kingdom). Line noise was removed using a Humbug mains noise eliminator (Quest Scientific, North Vancouver, Canada), and recordings were stored on a computer for off‐line analysis. While participants were required to maintain complete relaxation of the target FDI muscle during resting conditions, active conditions instead required contraction of FDI via index finger flexion to 5% of maximum (maintained using visual feedback of force production). Maximal voluntary contraction (MVC) force was assessed during index finger flexion, using a series of 3 s contractions separated by a 30 s break. The highest force level achieved was set as MVC.

### 2.3 TMS

Monophasic TMS pulses were applied to the left M1 using a figure‐of‐eight branding iron coil connected to two Magstim 200^2^ stimulators via a Bistim unit (Magstim, Dyfed, United Kingdom). The coil was held tangentially to the scalp at an angle of approximately 45° to the sagittal plane, at the location producing the largest stable response in the relaxed right FDI muscle. The current induced by stimulation was applied perpendicular to the assumed direction of the central sulcus with either a PA or AP orientation. The hot spot was co‐registered to the MNI‐ICBM152 brain template (Fonov et al., 2011) using a Brainsight neuronavigation system (Rogue Research Inc, Montreal, Canada) in each experimental session. Stimulation was applied at a rate of .2 Hz with a 10% jitter between trials to avoid anticipation of the stimulus. Resting motor threshold (RMT) was defined as the minimum intensity needed to evoke MEPs ≥ 50 μV in at least 5 of 10 consecutive trials during relaxation of the right FDI muscle. In contrast, active motor threshold (AMT) was defined as the minimum intensity that elicited MEPs of ≥200 μV in at least 5 of 10 consecutive trials, while participants maintained a light muscle contraction of 10% MVC. Stimulus intensity was expressed as a percentage of maximum stimulator output (MSO). At the start of each experimental session, the stimulus intensity producing the required test MEP amplitude (range: .5–1.0 mV in Experiments 1 and 3, 1.0–2.0 mV in Experiment 2; averaged over 15 trials) was assessed. The same intensity was then reapplied following application of iTMS to characterise changes in corticospinal excitability.

#### 2.3.1 SICF

SICF involved a subthreshold conditioning stimulus (CS) applied following a suprathreshold test stimulus (TS) at ISIs of 1.5 (SICF_1.5ms_) and 4.3 ms (SICF_4.3ms_), corresponding to the first and third SICF peaks (Delvendahl et al., 2014; Opie et al., 2018). Intensity of the CS was set at 90% RMT (for resting trials) or 90% AMT (for active trials), whereas the TS was set at the intensity required to produce an MEP of ~.5–1 mV in Experiments 1 and 3 and ~1–2 mV in Experiment 2 (when averaged over 15 trials). SICF at each time point was assessed using a single block of 45 trials (15 each of SICF_1.5_, SICF_4.3ms_ and TS), with the order of each stimulus condition pseudorandomised within a block. The same intensities were applied following the intervention.

### 2.4 Study design

#### 2.4.1 Experiment 1: PA‐ and AP‐iTMS in resting muscle

This experiment involved 16 young adults (mean age ± SD: 24.1 ± 4.9, 12 females) and sought to investigate how the direction of current applied during iTMS influences the neuroplastic response to the intervention. Within each session, iTMS was applied with a 1.5 ms ISI using either PA (PA iTMS) or AP (AP iTMS) coil orientation. The intensity of stimulation during iTMS was the same for both stimuli (Opie et al., 2021; Thickbroom et al., 2006) and was adjusted so that paired stimulation produced a response amplitude of ~1 mV (assessed over 15 trials before the intervention). Neuroplastic changes in corticospinal excitability were assessed by recording PA and AP MEPs and SICF before, 5 min after and 30 min after application of iTMS (Figure 1 and Table 1). All measures were applied during complete relaxation of FDI. Although previous studies have applied iTMS over 15 min (Long et al., 2017; Opie et al., 2018), the higher intensities required when using an AP coil orientation meant that coil heating precluded this duration of stimulation. Given that 10 min of iTMS has been previously shown to induce M1 plasticity (Sewerin et al., 2011) and to maximise the number of participants we were able to include, iTMS in Experiment 1 was therefore limited to 10 min of stimulation, resulting in application of 120 stimulus pairs (in contrast to the 180 pairs with conventional iTMS).

**TABLE 1:** iTMS parameters in each experiment.

	Pairs	Time (min)	Orientation	State during iTMS
Experiment 1 (*n* = 16)				
Session 1	120	10	PA	Rest
Session 2	120	10	AP	Rest
Experiment 2 (*n* = 17)				
Session 1	60	5	PA	Active
Session 2	60	5	AP	Active
Experiment 3 (*n* = 14)				
Session 1	60	5	AP	Rest
Session 2	60	5	AP	Active

Abbreviations: AP, anterior–posterior; iTMS, I‐wave periodicity repetitive transcranial magnetic stimulation; PA, posterior–anterior.

#### 2.4.2 Experiment 2: PA‐ and AP‐iTMS in active muscle

This experiment involved 17 young adults (mean age ± SD: 23.9 ± 4.9, 13 females) and sought to assess the influence of voluntary activation on the neuroplastic response to iTMS applied with different current directions. Each session again involved either PA iTMS or AP iTMS, with an ISI of 1.5 ms and intensity producing a 1 mV response (following paired stimulation over 15 trials). However, in contrast to Experiment 1, iTMS was applied during low‐level voluntary contraction (5% MVC) of FDI. While PA and AP MEPs and SICF were again recorded in resting muscle at pre and 5 min post time points, these measures were also repeated during voluntary activation of FDI (5% MVC) (Figure 1 and Table 1). As previous work has shown that voluntary activation increases the efficacy of neuroplasticity induction (Kujirai et al., 2006) and to reduce the generation of muscle fatigue (Kotan et al., 2015), the iTMS intervention was further shortened to 60 stimuli applied over 5 min.

#### 2.4.3 Experiment 3: AP‐iTMS in resting and active muscle

This experiment involved 14 young adults (mean age ± SD: 25.7 ± 5.6, 7 females) and sought to assess how activating the muscle during pre‐ and post‐intervention measurements influenced the response to 5 min of AP iTMS. Consequently, AP‐iTMS was applied during both rest and voluntary activation of FDI muscle (5% MVC), whereas pre‐ and post‐intervention PA and AP MEPs and SICF were only recorded in the resting state. Consistent with Experiment 2, iTMS involved 60 stimuli applied over 5 min, using an ISI of 1.5 ms and intensity set to produce a 1 mV response to paired stimulation.

### 2.5 Data analysis

MEP data were inspected visually and trials with muscle activity >20 μV peak‐to‐peak amplitude in the 100 ms prior to TMS were rejected. MEP amplitude recorded in each trial was then quantified peak to peak and expressed as a percentage of the baseline test MEP. For post‐intervention responses, previous work suggests that increased facilitation following iTMS correlates with the increased response to single pulse stimulation and that this relationship cancels the effects of iTMS on SICF if the post‐intervention single‐pulse MEPs are used to normalise post‐intervention SICF values (Cash et al., 2009). As Spearman's rank correlation test revealed a similar relationship within the data of the current study (Experiment 1: *rho* = .6, *P* = .02; Experiment 2: *rho* = .5, *P* = .03; Experiment 3: *rho* = .9, *P* < .01), individual post‐intervention SICF trials were instead expressed relative to the mean pre‐intervention single‐pulse MEP (Cash et al., 2009; Liao et al., 2022; Opie et al., 2021). MEP amplitudes recorded during iTMS were averaged over 10 consecutive stimuli, resulting in a total of 12 blocks in Experiment 1 and 6 blocks in Experiments 2 and 3. All responses during iTMS were expressed relative to the mean response amplitude from the first block.

### 2.6 Statistical analysis

All analyses were performed using PASW statistics software version 28 (SPSS; IBM, Armonk, NY, United States). Visual inspection and Kolmogorov–Smirnov tests revealed non‐normal (all *P* < .05), positively skewed distributions for all TMS data residuals. To facilitate investigation of non‐normally distributed data, generalised linear mixed models (GLMMs) were used to perform all TMS data analyses (Lo & Andrews, 2015; Puri & Hinder, 2022). Models were fitted using Gamma distributions, with identity link functions used for raw MEP amplitudes and log link functions used for responses expressed as a percentage (baseline‐normalised responses, normalised iTMS responses and baseline SICF) (Lo & Andrews, 2015; Puri & Hinder, 2022). All random subject effects (intercepts and slopes) were included, provided that the model converged (Barr et al., 2013). Model fit was optimised by testing different covariance structures, with the structure providing the best fit (assessed with the Bayesian Schwartz Criterion; BIC) within a model that was able to converge used in the final model. All main effects and interactions were performed using custom contrasts with Bonferroni correction, and significance was set at *P* < .05. All data are presented as estimated marginal means (EMM) and 95% confidence intervals (95% CI), whereas pairwise comparisons are presented as the estimated mean difference (EMD) and 95% CI.

#### 2.6.1 Experiment 1

Baseline characteristics were investigated by assessing the effects of iTMS session (resting PA iTMS and resting AP iTMS) and coil orientation (PA and AP) on stimulation intensities for RMT, AMT and TS and the effects of session on the stimulation intensity used for iTMS. Baseline TMS responses were investigated by assessing the effects of session and coil orientation on test MEP, SICF_1.5ms_ and SICF_4.3ms_, in addition to the effects of session on MEP amplitude during the first block of iTMS. Changes in responses as a result of iTMS were investigated by assessing the effects of session, coil orientation and time (5 and 30 min) on baseline‐normalised test MEP, SICF_1.5ms_ and SICF_4.3ms_. Responses during iTMS were investigated by assessing the effects of session and iTMS block (Blocks 2–12) on responses normalised to the first iTMS block.

#### 2.6.2 Experiment 2

Baseline characteristics were investigated by assessing the effects of session (active PA iTMS and active AP iTMS) and coil orientation on stimulation intensities for RMT, AMT, resting TS and active TS and the effects of session on the stimulation intensity used for iTMS. Baseline TMS responses were investigated by assessing the effects of session and coil orientation on the resting and active conditions of test MEP, SICF_1.5ms_ and SICF_4.3ms_, in addition to the effects of session on MEP amplitude during the first block of iTMS. Changes in responses due to iTMS were investigated by assessing the effects of session and coil orientation on the resting and active conditions of baseline‐normalised test MEP, SICF_1.5ms_ and SICF_4.3ms_. Responses during iTMS were investigated by assessing the effects of session and iTMS block (Blocks 2–6) on normalised iTMS responses.

#### 2.6.3 Experiment 3

Baseline characteristics were investigated by assessing the effects of session (resting AP iTMS and active AP iTMS) and coil orientation on stimulation intensities for RMT and TS and the effects of session on the stimulation intensity used for iTMS. Baseline TMS responses were investigated by assessing the effects of session and coil orientation on test MEP, SICF_1.5ms_ and SICF_4.3ms_, in addition to the effects of session on MEP amplitude during the first block of iTMS. Changes in responses due to iTMS were investigated by assessing the effects of session and coil orientation on baseline‐normalised test MEP, SICF_1.5ms_ and SICF_4.3ms_. Responses during iTMS were investigated by assessing the effects of session and iTMS block (Blocks 2–6) on normalised iTMS responses.

## 3 RESULTS

Although 31 participants completed all sessions, one participant withdrew from Experiment 3, as they experienced a mild headache following completion of the first session (in resting muscle). While these participant's data were able to be included within analysis of the resting condition for Experiment 3, the cohort for the active condition was reduced to *n* = 13. No other adverse reactions were reported. A total of 4.2% of MEP trials in resting muscle were removed due to muscle activity across all participants in the three experiments. Baseline stimulus characteristics and TMS responses for each experiment are presented in Table 2, with the stimulus characteristics reported in detail within supporting information results. Furthermore, normalised MEP and SICF data (% baseline) are described in detail below, with absolute data provided in the supporting information results.

**TABLE 2:** Baseline characteristics, corticospinal and intracortical responses (EMM [95% CI; lower, upper]) for each experiment.

		iTMS		MEP		RMT	AMT	MEP_.5‐1mV_	MEP_1‐2mV_	iTMS	MEP_.5‐1mV_	MEP_1‐2mV_	SICF_1.5ms_	SICF_4.3ms_
		Orientation	State	Orientation	State	(% MSO)	(% MSO)	(% MSO)	(% MSO)	(% MSO)	(mV)	(mV)	(% baseline)	(% baseline)
**Experiment 1**														
(*n* = 16)	Session 1	PA	Rest	PA	Rest	47.3 [43.5, 51.0]	45.0 [41.9, 48.1]	54.5 [50.1, 58.9]		49.8 [45.8, 53.7]	.67 [.51, .75]	–	208.7 [175.9, 247.5]	111.1 [95.7, 129.0]
		PA	Rest	AP	Rest	60.1 [56.4, 63. 9]*	58.2 [55.0, 61.3]*	73.9 [69.5, 78.3]*			.65 [.54, .77]	–	168.0 [141.7, 199.3]	115.9 [99.8, 134.7]
	Session 2	AP	Rest	PA	Rest	47.6 [43.9, 51.4]	45.0 [41.9, 48.1]	57.2 [52.8, 61.6]		65.8 [61.8, 69.8]**	.70 [.58, .83]	–	187.5 [157.9, 222.5]	119.6 [102.9, 139.0]
		AP	Rest	AP	Rest	60.4 [56.6, 64.1]*	56.8 [53.7, 60.0]*	73.7 [69.3, 78.1]*			.65 [.53, .77]	–	180.5 [152.2, 214.2]	116.1 [100.0, 134.8]
**Experiment 2**														
(*n* = 17)	Session 1	PA	Active	PA	Rest	45.3 [42.2, 48.4]	43.2 [40.1, 46.3]	–‐	60.7 [57.8, 63.5]	41.5 [38.4, 44.7]	–‐	1.36 [1.14, 1.58]	126.7 [109.8, 146.3]	106.1 [94.1, 119.7]
		PA	Active	PA	Active			–	51.2 [48.4, 54.1]		–	1.75 [1.51, 1.99]	201.3 [164.7, 245.9]	102.5 [90.2, 116.6]
		PA	Active	AP	Rest	59.2 [55.5, 63.0]*	57.1 [54.0, 60.2]*	–	79.5 [79.6, 82.5]*		–	1.31 [1.09, 1.53]	127.3 [110.3, 147.0]	100.5 [89.1, 113.3]
		PA	Active	AP	Active			–	65.8 [62.9, 68.7]*		–	1.55 [1.31, 1.78]	191.6 [156.8, 234.1]	101.1 [89.0, 115.0]
	Session 2	AP	Active	PA	Rest	44.0 [41.0, 47.1]	42.2 [39.2, 45.3]	–	59.6 [56.8, 62.5]	56.9 [53.7, 60.0]**	–	1.23 [1.01, 1.44]**	132.9 [115.1, 153.5]	113.0 [100.2, 127.5]
		AP	Active	PA	Active			–	50.0 [47.1, 52.8]		–	1.33 [1.10, 1.56]**	232.9 [190.6, 284.6]	106.8 [93.9, 121.4]
		AP	Active	AP	Rest	58.5 [54.8, 62.3]*	55.8 [52.7, 58.8]*	–	78.7 [75.8, 81.7]*		–	1.14 [.92, 1.36]**	124.6 [107.9, 143.8]	102.7 [91.0, 115.8]
		AP	Active	AP	Active			–	65.7 [62.8, 68.6]*		–	1.31 [1.08, 1.54]**	215.3 [176.2, 263.1]	115.4 [101.5, 131.2]
**Experiment 3**														
(*n* = 14)	Session 1	AP	Rest	PA	Rest	50.7 [46.7, 54.7]	–	60.5 [55.9, 65.1]	–	72.1 [65.8, 78.5]	.64 [.51, .76]	–	240.2 [200.0, 288.4]	133.9 [104.7, 171.4]
		AP	Rest	AP	Rest	66.4 [61.4, 71.4]*	–	79.7 [74.0, 85.4]*	–		.56 [.44, .68]	–	222.6 [185.3, 267.5]	130.7 [102.0, 167.5]
	Session 2	AP	Active	PA	Rest	50.9 [46.8, 54.9]	–	60.6 [55.9, 65.2]	–	63.9 [57.3, 70.6]**	.77 [.63, .91]	–	219.7 [181.8, 265.4]	138.7 [107.6, 178.7]
		AP	Active	AP	Rest	68.5 [63.3, 73.6]*	–	80.9 [75.1, 86.7]*	–		.65 [.52, .78]	–	210.1 [173.7, 254.0]	153.5 [119.0, 198.1]

Abbreviations: AMT, active motor threshold; AP, anterior–posterior; iTMS, I‐wave periodicity repetitive transcranial magnetic stimulation; MEP, motor‐evoked potential; % MSO, %maximum stimulator output; PA, posterior–anterior; RMT, resting motor threshold; SICF, short‐interval intracortical facilitation.

* *P* < .05 compared to PA MEPs.

** *P* < .05 compared to Session 1.

### 3.1 Experiment 1

Baseline test MEP amplitude did not differ between sessions (*F*_1, 925_ = .4, *P* = .5) or coil orientations (*F*_1, 925_ = .1, *P* = .8), and there was no interaction between factors (*F*_1, 925_ = .8, *P* = .4). Baseline SICF_1.5ms_ did not vary between sessions (*F*_1, 940_ = .1, *P* = .8) or coil orientations (*F*_1, 940_ = 2.6, *P* = .1), and there was no interaction between factors (*F*_1, 940_ = 2.5, *P* = .1). Baseline SICF_4.3ms_ did not vary between sessions (*F*_1, 941_ = .3, *P* = .6) or coil orientations (*F*_1, 941_ = .01, *P* = .9), and there was no interaction between factors (*F*_1, 941_ = .4, *P* = .5). The first block of iTMS revealed no differences between sessions (*F*_1, 318_ = .3, *P* = .6).

**FIGURE 2:**
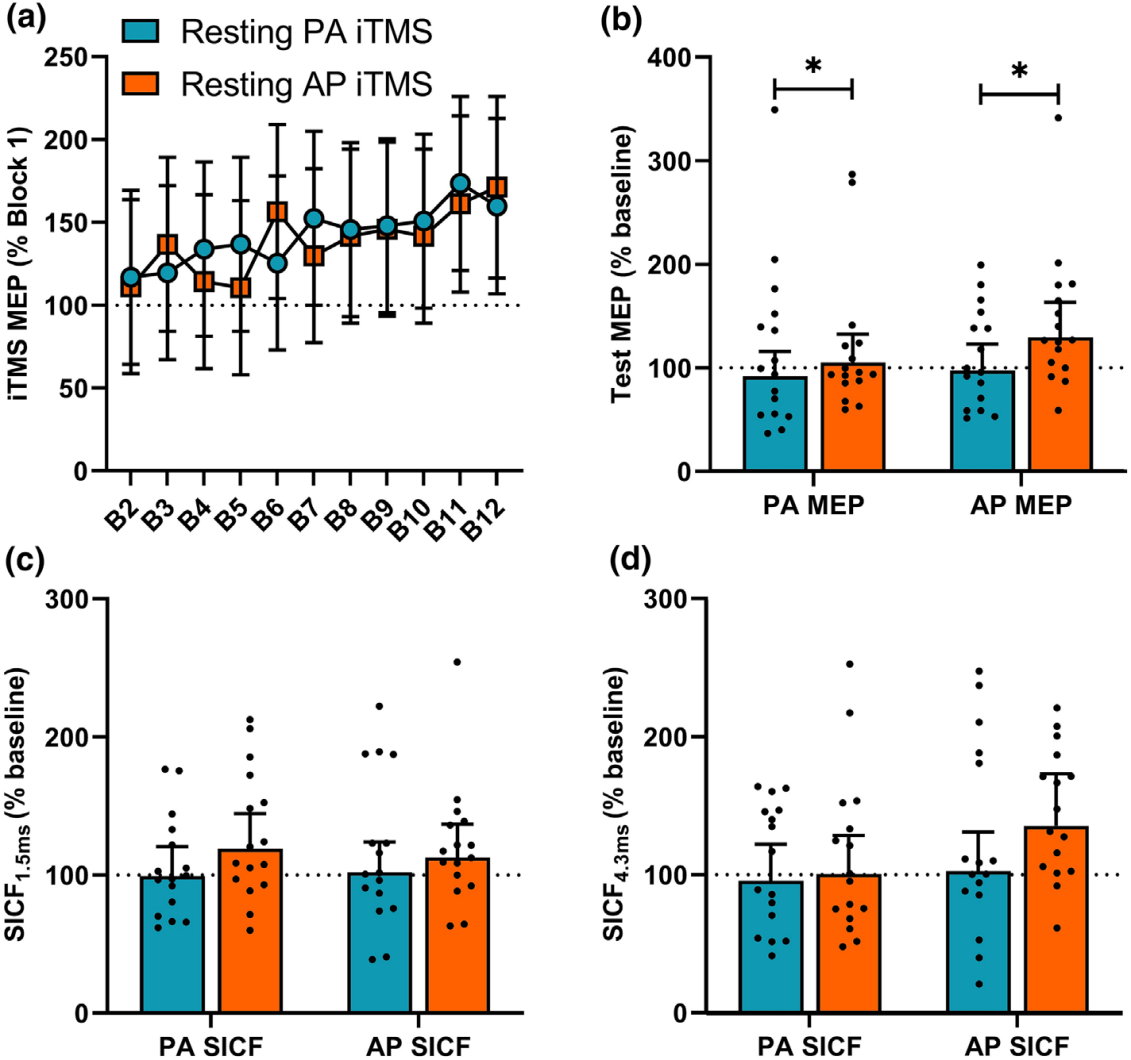
Corticospinal and intracortical excitability changes induced by PA and AP iTMS in resting muscle (Experiment 1). (a) Responses during PA iTMS (blue circles) and AP iTMS (orange squares) in the resting muscle were averaged over 10 consecutive MEP trials, with Blocks 2–12 normalised to Block 1. (b) Baseline‐normalised PA and AP MEP amplitudes following iTMS. Baseline‐normalised PA and AP SICF_1.5ms_ (c) and SICF_4.3ms_ (d) following iTMS. *P < .05.

Figure 2a shows changes in MEP amplitude during iTMS, normalised to the first block. While no differences were shown between sessions (*F*_1, 3,420_ = .1, *P* = .7), MEP amplitude varied over blocks (*F*_10, 3,420_ = 2.0, *P* = .03), although post hoc comparisons revealed no differences (all *P* > .05). There was no interaction between factors (*F*_10, 3,420_ = 1.0, *P* = .5).

Figure 2b shows test MEP amplitude following iTMS. While there were no differences between coil orientations (*F*_1, 1,881_ = 1.6, *P* = .2) or times (*F*_1, 1,881_ = .4, *P* = .5), MEP amplitude varied between sessions (*F*_1, 1,881_ = 4.0, *P* < .05), with post hoc revealing increased responses following resting AP iTMS relative to resting PA iTMS (EMD = 22.0% [.0, 44.0], *P* < .05). There were no significant interactions between factors (all *P* > .05).

Figure 2c,d shows SICF responses following iTMS. SICF_1.5ms_ (Figure 2c) did not vary between sessions (*F*_1, 1,883_ = 2.6, *P* = .1), coil orientations (*F*_1, 1,883_ = .02, *P* = .9) or time points (*F*_1, 1,883_ = .05, *P* = .8), and no interaction between factors (all *P* > .05). SICF_4.3ms_ (Figure 2d) did not vary between sessions (*F*_1, 1,874_ = 2.3, *P* = .1), coil orientations (*F*_1, 1,874_ = 2.8, *P* = .1) or time points (*F*_1, 1,874_ = .07, *P* = .8) and no interaction between factors (all *P* > .05).

Given that there were differences in PA and AP iTMS intensities (Table 2), we performed two additional analyses to determine if a greater AP iTMS intensity contributed to a greater iTMS response. First, we performed a Spearman's rank‐order correlation of the difference in PA and AP iTMS intensities in each participant with the difference in the magnitude of the response (MEP and SICF) after PA and AP iTMS. This was performed to determine if larger differences in PA and AP iTMS intensities resulted in greater differences in post iTMS effects. This analysis showed no correlation between the difference in AP‐PA iTMS intensity (% MSO) and the post iTMS difference in MEP (PA MEP_.5‐1mV_ difference, ρ = −.103, *P* = .703; AP MEP_.5‐1mV_ difference, ρ = −.065, *P* = .811) or SICF (PA SICF_1.5_ difference, ρ = .055, *P* = .841; AP SICF_1.5_ difference, ρ = .04, *P* = .883; PA SICF_4.3_ difference, ρ = −.256, *P* = .339; AP SICF_4.3_ difference, ρ = −.001, *P* = .996). Second, we performed a sub‐sample analysis where participants were separated into those with small (≤15% MSO difference; range 4–15% MSO; nine participants) or large (>15% MSO; range 18–29% MSO; seven participants) differences in PA and AP iTMS intensity and then compared the difference in the response to PA and AP iTMS between groups (Table 3). If differences in PA and AP iTMS intensities were contributing to the greater responses following AP iTMS, then we would expect to see greater differences in the response in participants with large differences in AP‐PA iTMS intensities (>15% MSO). GLMM analysis showed that there were no significant differences in any MEP or SICF measure between groups (Table 3; all *P* > .05).

**TABLE 3:** Differences in post‐intervention MEP amplitude and SICF between AP iTMS and PA iTMS for participants whose AP‐PA iTMS stimulation intensity ≤15% MSO (*n* = 9) and >15% MSO (*n* = 7).

Measure (% baseline)		Differences between AP iTMS and PA iTMS		
		AP‐PA iTMS % MSO ≤ 15	AP‐PA iTMS % MSO > 15	*P* value
PA	MEP_.5‐1mV_	6.1 [−32.8, 45.0]	−17.6 [−61.7, 26.5]	.403
AP	MEP_.5‐1mV_	28.0 [−18.2, 74.3]	2.9 [−49.5, 55.3]	.453
PA	SICF_1.5ms_	−53.8 [−122.5, 14.8]	−34.9 [−112.7, 43.0]	.701
AP	SICF_1.5ms_	−7.5 [−81.6, 66.6]	−32.4 [−116.5, 51.6]	.641
PA	SICF_4.3ms_	16.3 [−24.1, 56.8]	3.3 [−42.6, 49.1]	.654
AP	SICF_4.3ms_	52.0 [−3.1, 107.2]	−13.8 [−76.3, 48.8]	.113

*Note*: Data are presented as EMM (95% CI; lower, upper), with positive values indicating larger responses for AP iTMS.

### 3.2 Experiment 2

Baseline resting test MEP amplitude did not differ between sessions (*F*_1, 987_ = 2.4, *P* = .1) or coil orientations (*F*_1,987_ = .5, *P* = .5), and there was no interaction between factors (*F*_1, 987_ = .1, *P* = .8). In contrast, while baseline active test MEP amplitude did not vary between coil orientations (*F*_1, 1,008_ = 1.1, *P* = .3) and had no interaction between factors (*F*_1, 1,008_ = 1.6, *P* = .2), responses varied between sessions (*F*_1, 1,008_ = 9.5, *P* = .002), with comparisons showing increased responses during the active PA iTMS session relative to responses during active AP iTMS (EMD = .3 mV [.1, .5], *P* = .002). Baseline resting SICF_1.5ms_ did not vary between sessions (*F*_1, 1,009_ = .04, *P* = .8) or coil orientations (*F*_1, 1,009_ = .2, *P* = .6), and there was no interaction between factors (*F*_1, 1,009_ = .6, *P* = .5). Baseline active SICF_1.5ms_ did not differ between sessions (*F*_1, 1,004_ = 3.6, *P* = .06) or coil orientations (*F*_1, 1,004_ = .9, *P* = .4), and there was no interaction between factors (*F*_1, 1,004_ = .1, *P* = .7). Baseline resting SICF_4.3ms_ did not vary between sessions (*F*_1, 987_ = .6, *P* = .4) or coil orientations (*F*_1, 987_ = 1.8, *P* = .2), and there was no interaction between factors (*F*_1, 987_ = .3, *P* = .6). Baseline active SICF_4.3ms_ did not differ between sessions (*F*_1, 1,027_ = 2.1, *P* = .1) or coil orientations (*F*_1, 1,027_ = .3, *P* = .6), and there was no interaction between factors (*F*_1, 1,027_ = 1.1, *P* = .3). MEP amplitude during the first block of iTMS showed no differences between sessions (*F*_1, 338_ = 2.0, *P* = .2).

**FIGURE 3:**
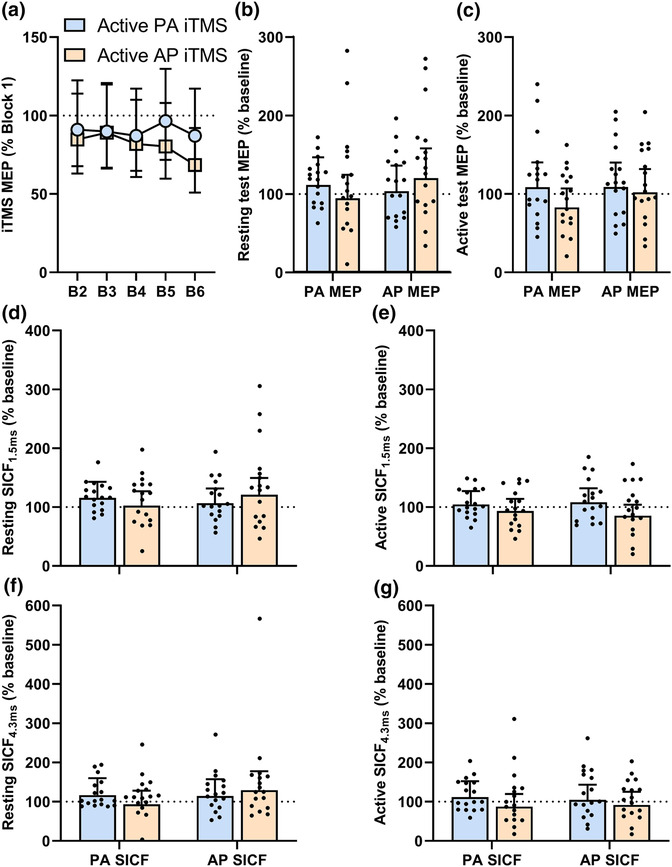
Corticospinal and intracortical excitability changes induced by PA and AP iTMS in active muscle (Experiment 2). (a) Responses during PA iTMS (light blue circles) and AP iTMS (light orange squares) in active muscles were averaged over six consecutive MEP trials, with Blocks 2–6 normalised to Block 1. (b) Baseline‐normalised PA and AP MEP amplitudes the resting muscle following iTMS. (c) Baseline‐normalised PA and AP MEP amplitudes the active muscle following iTMS. Baseline‐normalised PA and AP SICF_1.5ms_ in the resting (d) and active (e) muscle following iTMS. Baseline‐normalised PA and AP SICF_4.3ms_ in the resting (d) and active (e) muscle following iTMS.

Figure 3a shows changes in MEP amplitude during iTMS in an active muscle. There were no differences between sessions (*F*_1, 1,676_ = 1.5, *P* = .2) or blocks (*F*_4, 1,676_ = .6, *P* = .6), and there was no interaction between factors (*F*_4, 1,676_ = 1.0, *P* = .4).

Figure 3b,c shows the resting and active test MEP amplitude following iTMS. While resting test MEP amplitude (Figure 3b) did not differ between sessions (*F*_1, 996_ = .004, *P* = 1) or coil orientations (*F*_1, 996_ = .5, *P* = .5), there was an interaction between factors (*F*_1, 996_ = 3.9, *P* < .05). However, post hoc comparisons revealed no differences (all *P* > .05). For active test MEP amplitude (Figure 3c), there were no differences between sessions (*F*_1, 1,031_ = 2.1, *P* = .1) or coil orientations (*F*_1, 1,031_ = .83, *P* = .4), and there was no interaction between factors (*F*_1, 1,031_ = 1.8, *P* = .2).

Figure 3d–g shows the resting and active responses for SICF following iTMS. While resting SICF_1.5ms_ (Figure 3d) did not vary between sessions (*F*_1, 1,010_ = .002, *P* = 1) or coil orientations (*F*_1, 1,010_ = .2, *P* = .7), there was an interaction between factors (*F*_1, 1,010_ = 4.1, *P* = .04). However, post hoc did not show any differences (all *P* > .15). While active SICF_1.5ms_ (Figure 3e) did not differ between coil orientations (*F*_1, 1,016_ = .1, *P* = .8), responses varied between sessions (*F*_1, 1,016_ = 3.9, *P* = .049). However, post hoc analysis did not show a difference (*P* = .052). There was no interaction between factors (*F*_1, 1,016_ = 1.2, *P* = .3). Resting SICF_4.3ms_ (Figure 3f) did not vary between sessions (*F*_1, 975_ = .1, *P* = .7) or coil orientations (*F*_1, 975_ = 1.2, *P* = .3), and there was no interaction between factors (*F*_1, 975_ = 3.8, *P* = .052). Lastly, active SICF_4.3ms_ (Figure 3g) did not differ between sessions (*F*_1, 998_ = 1.9, *P* = .2) or coil orientations (*F*_1, 998_ = .004, *P* = .5), and there was no interaction between factors (*F*_1, 998_ = .4, *P* = .5).

### 3.3 Experiment 3

Baseline test MEP amplitude did not vary between sessions (*F*_1, 755_ = 3.3, *P* = .07) or coil orientations (*F*_1, 755_ = 2.6, *P* = .1), and there was no interaction between factors (*F*_1, 755_ = .2, *P* = .6). Baseline SICF_1.5ms_ did not vary between sessions (*F*_1, 786_ = .7, *P* = .4) or coil orientations (*F*_1, 786_ = .5, *P* = .5), and there was no interaction between factors (*F*_1, 786_ = .06, *P* = .8). Baseline SICF_4.3ms_ did not differ between sessions (*F*_1, 781_ = .7, *P* = .4) or coil orientations (*F*_1, 781_ = .1, *P* = .7), and there was no interaction between factors (*F*_1, 781_ = .6, *P* = .4). In contrast, MEP amplitude during the first block of iTMS differed between sessions (*F*_1, 268_ = 5.5, *P* = .02), with comparisons showing increases in the responses during the active AP iTMS relative to resting AP iTMS (EMD = .5 mV [.1, 1.0], *P* = .02).

**FIGURE 4:**
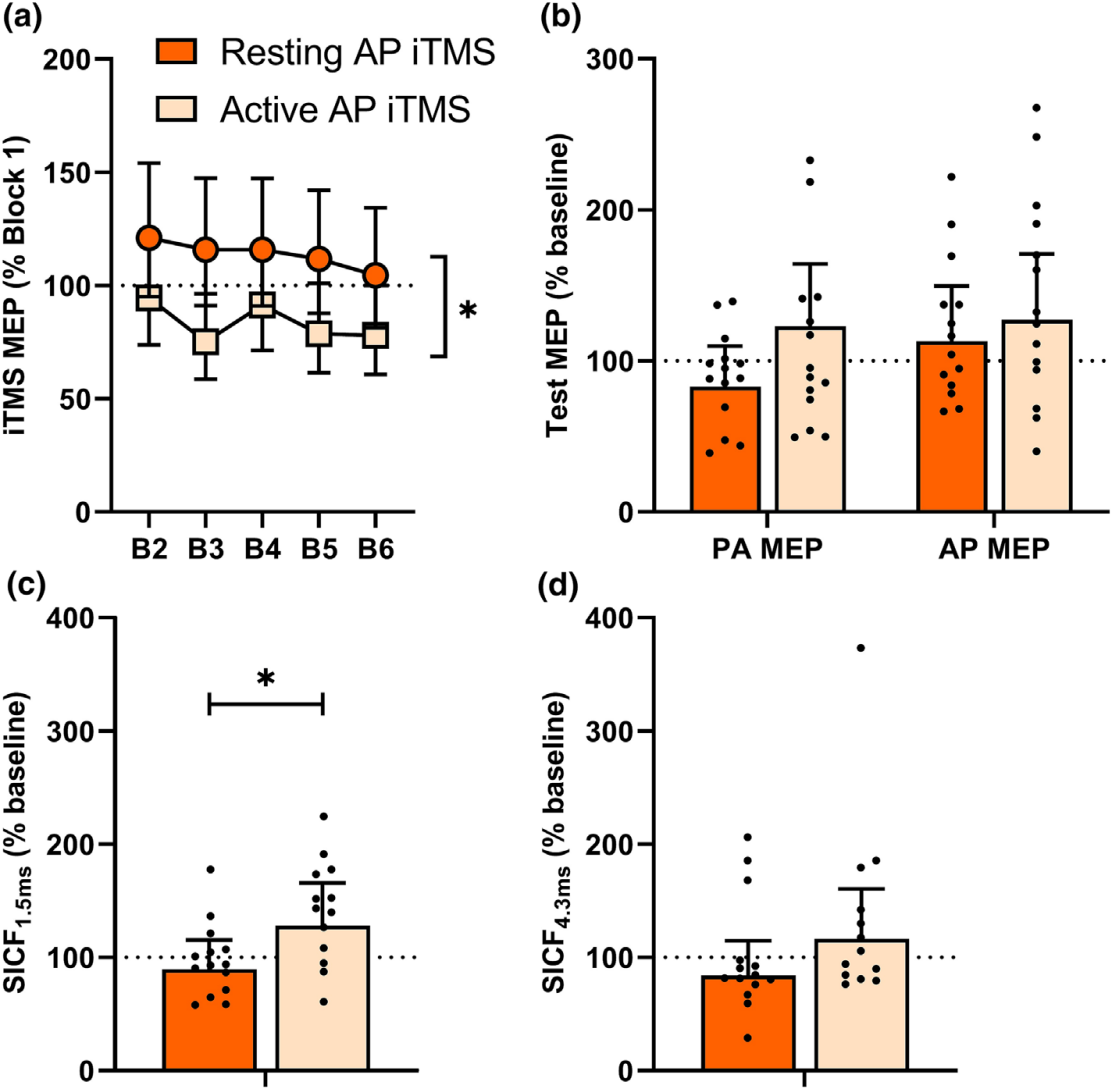
Corticospinal and intracortical excitability changes by AP iTMS in resting and active muscle (Experiment 3). (a) Normalised responses during AP iTMS in resting muscle (orange squares) and AP iTMS in active muscle (light orange squares). (b) Baseline‐normalised PA and AP MEP amplitudes following iTMS. Baseline‐normalised PA and AP SICF_1.5ms_ (c) and SICF_4.3ms_ (d) following iTMS. *P < .05.

Figure 4a shows changes in MEP amplitude during resting and active AP iTMS. While there were no differences between blocks (*F*_4, 1,305_ = .8, *P* = .5), MEP amplitude differed between sessions (*F*_1, 1,305_ = 10, *P* = .001), with post hoc revealing increased responses during resting AP iTMS relative to active AP iTMS (EMD = 30.4% [11.5, 49.3], *P* = .002). There was no interaction between factors (*F*_4, 1,305_ = .6, *P* = .7).

Figure 4b shows changes in MEP amplitude following iTMS. MEP amplitude did not vary between sessions (*F*_1, 751_ = 3.7, *P* = .06) or coil orientations (*F*_1, 751_ = 1.7, *P* = .2), and there was no interaction between factors (*F*_1, 751_ = 1.9, *P* = .2).

Figure 4c,d shows changes in SICF following iTMS. While SICF_1.5ms_ (Figure 4c) did not differ between coil orientations (*F*_1, 782_ = .3, *P* = .6), responses varied between sessions (*F*_1, 782_ = 6.9, *P* = .009), with post hoc revealing increased responses after active AP iTMS relative to resting AP iTMS (EMD = 38.4% [8.0, 68.8], *P* = .013). There was no interaction between factors (*F*_1, 782_ = .4, *P* = .5). For SICF_4.3ms_ (Figure 4d), responses did not vary between sessions (*F*_1, 780_ = 3.8, *P* = .052) or coil orientations (*F*_1, 780_ = .37, *P* = .5) and no interaction between factors (*F*_1, 780_ = .8, *P* = .4).

## 4 DISCUSSION

An extensive body of literature has established the ability of TMS current direction to influence the intracortical circuits recruited by stimulation. Within the current study, we investigated if this feature of TMS could be utilised to manipulate which intracortical circuits contribute to plasticity induction in M1. To achieve this aim, we completed a series of experiments involving a shortened iTMS intervention, within which current direction and muscle activity state were systematically modified, and the influence on both corticospinal and intracortical excitability was examined. Our findings show that AP iTMS produced greater changes in corticospinal excitability than PA iTMS after the intervention, and muscle activation reduced the effectiveness of AP iTMS on corticospinal excitability during the intervention. However, there were no consistent changes in intracortical excitability (as tested with SICF) after the intervention with either PA or AP iTMS in resting or active muscle.

### 4.1 Current direction influences the response to iTMS in resting muscle

Previous work using iTMS has reported reliable potentiation of MEP amplitude both during and after the intervention (for review, see Kidgell et al., 2016; Sasaki et al., 2023), which has been suggested to reflect the induction of neuroplastic changes in M1 (di Lazzaro et al., 2007). Using a shorter protocol (10 min) than most previous studies (15–30 min), we demonstrated a small change in MEP amplitude during the intervention (~130%, Figure 2b) that was similar to that observed in a previous study that used a 10 min iTMS intervention (Sewerin et al., 2011). However, the magnitude of these effects was less than the facilitation (~200%) that is often seen following conventional iTMS (for review, see Kidgell et al., 2016). In addition, SICF was largely unaffected by the intervention, which contrasts previous studies that applied 15 min of iTMS with a PA current (Sasaki et al., 2023). One explanation for this reduced efficacy is the lower number of stimuli that were applied during the intervention, which may have resulted in a level of activation that was below the dose required to achieve robust neuroplastic effects. Our decision to reduce the number of stimuli during iTMS was primarily based on the need to avoid TMS coil overheating due to the increased TMS intensity required to activate AP circuits, with the knowledge that previous studies have shown a plateau of MEP facilitation after 10 min of iTMS (for review, see Kidgell et al., 2016) and a demonstration of MEP facilitation following a 10 min iTMS intervention (Sewerin et al., 2011). Consequently, it will be important for future work to examine how dosing influences the response to AP iTMS.

Despite the reduced magnitude of effect with a 10 min iTMS intervention, it is interesting to note that Experiment 1 revealed a sensitivity of iTMS to current direction. Specifically, post‐intervention changes in MEP amplitude were greater when iTMS was applied with AP compared with PA stimulation. However, this effect was not dependent on the current direction used to record post‐intervention MEPs, as AP iTMS produced greater changes in both PA and AP MEPs compared with PA iTMS. One explanation for this lack of specificity is that the increased stimulus intensities required for AP iTMS activated a neuronal pool that included elements able to be recruited by both PA and AP stimulation (when recording post‐intervention MEPs). This is supported by our data showing that the average stimulus intensity applied during AP iTMS represented a substantially higher proportion of RMT for PA (138%) than AP (109%) current. To further examine this difference, we performed two additional analyses to examine the effect of differences in PA and AP iTMS intensity on the post iTMS response. We found no correlation between the difference in AP‐PA iTMS intensity and the difference in the post iTMS response in individual participants. Furthermore, using a sub‐sample analysis, there was no difference in the post iTMS response when separating participants who had small (≤15% MSO) or large (>15% MSO) differences in PA and AP iTMS intensity (Table 3). These findings indicate that differences in intensity between PA and AP iTMS did not influence the response to iTMS.

### 4.2 Muscle activation did not improve the efficacy of iTMS

Previous work has shown that TMS current direction and muscle activation state can be used to influence neuroplasticity induction. For example, relative to PA TMS in resting or active muscle, the response to PAS is increased when applied in an active muscle using subthreshold AP TMS (Kujirai et al., 2006). This was suggested to reflect more isolated modulation of the late I‐wave circuits, which have been strongly implicated in the response to many plasticity‐inducing paradigms, including PAS (di Lazzaro et al., 2012). Within Experiment 2, we therefore reasoned that it may be possible to manipulate which I‐waves contribute to the iTMS response by applying stimulation in an active muscle. However, muscle activation abolished any effects of iTMS on MEPs and SICF, both during and after the intervention. Furthermore, this occurred irrespective of how post‐intervention responses were assessed (i.e., current direction or activation state). Like Experiment 1, the reduced number of stimuli (60 pairs over 5 min) applied during iTMS in an active muscle may have contributed to this outcome. However, this was necessary to avoid muscle fatigue from a prolonged contraction. Furthermore, this duration is similar to a previous study that induced M1 plasticity with 50 pairs of stimuli during muscle activation with PAS (Kujirai et al., 2006). Nonetheless, data from Experiment 3 (Resting vs. Active AP iTMS over 5 min) showed a reduced MEP facilitation during active compared with resting AP iTMS when using the same number of stimuli (60 pairs of stimuli over 5 min), suggesting that muscle activation influences the change in corticospinal excitability during (but not after) AP iTMS.

Consistent with the reduction in corticospinal excitability during iTMS in an active muscle, previous work applying theta burst stimulation (TBS) reported that voluntary activation during stimulation removed neuroplastic effects of the intervention (Huang et al., 2008). One explanation proposed by the authors was that contraction and TBS targeted the same synapses, the activity of which were saturated by activity associated with contraction. Consequently, the input associated with TBS was unable to evoke additional activation that would be required to modify the synapse (Huang et al., 2008). It is therefore possible that the sensitivity of iTMS to muscle activation may also involve similar mechanisms. Furthermore, it highlights the likely importance of the afferent stimulus involved in PAS for mediating a beneficial effect of contraction on plasticity induction. While the specific mechanisms remain unclear, it could be possible that the afferent volley with PAS provides an interruption to ongoing synaptic input (associated with contraction) that is sufficient for TMS‐associated activation to also influence the synapse. Additionally, priming effects of the afferent input may alter excitation levels in an extended neuronal pool, such that the pool responding to TMS extends beyond the pool involved with voluntary activation. One question that stems from these possibilities is whether the combination of afferent input, iTMS and contraction would facilitate greater manipulation of the circuits targeted by iTMS. It will therefore be interesting to investigate this possibility in future work.

### 4.3 Pre‐ and post‐interventional contraction did not influence the response to AP iTMS

In addition to the direct influence of concurrent activation during iTMS, the results of Experiment 2 could also be explained by the contractions that occurred before and after the intervention (i.e., for measuring MEPs/SICF in active muscle). For example, voluntary activation both prior to and following application of TBS has been shown to invert (Iezzi et al., 2008) or abolish (Gentner et al., 2008) effects on corticospinal excitability, possibly via higher order plasticity mechanisms such as metaplasticity (for review, see Ziemann & Siebner, 2008) and de‐potentiation/de‐depression (for review, see Zhou & Poo, 2004). We assessed this possibility in Experiment 3, where the response to AP iTMS applied in both resting and active muscle was assessed after iTMS using resting state measures only. In contrast to Experiment 2, there was a facilitation of SICF_1.5ms_ by AP iTMS in active muscle (Figure 4c). However, this was relative to the resting state only and was not influenced by current direction. Furthermore, consistent with Experiment 2, MEPs after active iTMS remained unchanged. Consequently, it seems unlikely that muscle activation during the intervention had any meaningful effect on the broad neuroplastic capacity of AP iTMS or on its ability to selectively modulate different intracortical circuits.

### 4.4 Limitations and other considerations

In order to examine the effect of TMS coil orientation and muscle activation on the neuroplastic response to iTMS, we had to use a modified iTMS protocol that was specific to each experimental session. In particular, we used a shortened protocol of 10 min (120 paired stimuli) in Experiment 1 to avoid coil overheating during AP iTMS, and we used a 5‐min protocol (60 paired stimuli) in Experiments 2 and 3 to avoid fatigue during muscle activation. This resulted in a different number of paired stimuli for each experiment, making it difficult to directly compare the magnitude of neuroplastic effects between experiments. Furthermore, the iTMS approach was based on a standard ISI of 1.5 ms, which reflects the periodicity of high frequency descending volleys with TMS (Day et al., 1989). However, there can be substantial variability in the latency of the early I‐wave response in different individuals, and a greater iTMS response can be achieved if the iTMS ISI is adjusted to the individual I‐wave periodicity (Sewerin et al., 2011). Unfortunately, it is not possible with current techniques to directly quantify the effectiveness of iTMS on I‐wave periodicity during the intervention, potentially resulting in differences in the effectiveness of iTMS and greater variability between participants. Finally, TMS at the intensities used here is likely to activate a combination of I‐waves, and voluntary contraction is known to increase the number and size of I‐waves (di Lazzaro et al., 1998). We found that AP iTMS increased both PA and AP MEPs when applied in a resting but not active muscle, so it is possible that AP iTMS activates specific cortical circuits, and these are more amenable to stimulation in a resting muscle. However, as far as we are aware, it is not known how voluntary contraction influences I‐wave characteristics for TMS with an AP current.

In conclusion, the current study aimed to alter which intracortical circuits were targeted by iTMS, by manipulating current direction and activation state. While this approach identified increased corticospinal excitability with AP iTMS compared with PA iTMS when applied at rest, this effect involved both PA and AP I‐wave circuits. Furthermore, effects of AP iTMS on corticospinal excitability were removed when iTMS was applied in an active muscle. Although these findings do not support the concept of targeted I‐wave modulation, further investigation is required to assess how dosing and afferent input may modulate this outcome.

## AUTHOR CONTRIBUTIONS

**Ryoki Sasaki:** Data curation; formal analysis; funding acquisition; investigation; methodology; resources; writing—original draft; writing—review and editing. **Wei‐Yeh Liao:** Data curation; formal analysis; investigation; methodology; writing—review and editing. **George M. Opie:** Conceptualization; funding acquisition; investigation; methodology; project administration; resources; supervision; writing—original draft; writing—review and editing. **John G. Semmler:** Conceptualization; funding acquisition; investigation; methodology; project administration; resources; supervision; writing—review and editing.

## CONFLICT OF INTEREST STATEMENT

The authors declare no conflict of interest.

### PEER REVIEW

The peer review history for this article is available at https://publons.com/publon/10.1111/ejn.16099.

## Supporting information


**Table S1.** Absolute MEP amplitude and SICF data at each time point for all three experiments (mean ± standard deviation).

## Data Availability

Data from this study will be made available to qualified investigators upon reasonable request to the corresponding author.

## References

[ejn16099-bib-0001] Barker, A. T. , Jalinous, R. , & Freeston, I. L. (1985). Non‐invasive magnetic stimulation of human motor cortex. Lancet, 1, 1106–1107. 10.1016/S0140-6736(85)92413-4 2860322

[ejn16099-bib-0002] Barr, D. J. , Levy, R. , Scheepers, C. , & Tily, H. J. (2013). Random effects structure for confirmatory hypothesis testing: Keep it maximal. Journal of Memory and Language, 68, 255–278. 10.1016/j.jml.2012.11.001 PMC3881361 24403724

[ejn16099-bib-0004] Cash, R. F. , Benwell, N. M. , Murray, K. , Mastaglia, F. L. , & Thickbroom, G. W. (2009). Neuromodulation by paired‐pulse TMS at an I‐wave interval facilitates multiple I‐waves. Experimental Brain Research, 193, 1–7. 10.1007/s00221-008-1590-7 18850091

[ejn16099-bib-0005] Day, B. L. , Dressler, D. , Maertens de Noordhout, A. , Marsden, C. D. , Nakashima, K. , Rothwell, J. C. , & Thompson, P. D. (1989). Electric and magnetic stimulation of human motor cortex: Surface EMG and single motor unit responses. The Journal of Physiology, 412, 449–473. 10.1113/jphysiol.1989.sp017626 2489409 PMC1190586

[ejn16099-bib-0006] Delvendahl, I. , Lindemann, H. , Jung, N. H. , Pechmann, A. , Siebner, H. R. , & Mall, V. (2014). Influence of waveform and current direction on short‐interval intracortical facilitation: A paired‐pulse TMS study. Brain Stimulation, 7, 49–58. 10.1016/j.brs.2013.08.002 24075915

[ejn16099-bib-0007] di Lazzaro, V. , Oliviero, A. , Mazzone, P. , Insola, A. , Pilato, F. , Saturno, E. , Accurso, A. , Tonali, P. , & Rothwell, J. C. (2001). Comparison of descending volleys evoked by monophasic and biphasic magnetic stimulation of the motor cortex in conscious humans. Experimental Brain Research, 141, 121–127. 10.1007/s002210100863 11685416

[ejn16099-bib-0008] di Lazzaro, V. , Oliviero, A. , Saturno, E. , Pilato, F. , Insola, A. , Mazzone, P. , Profice, P. , Tonali, P. , & Rothwell, J. C. (2001). The effect on corticospinal volleys of reversing the direction of current induced in the motor cortex by transcranial magnetic stimulation. Experimental Brain Research, 138, 268–273. 10.1007/s002210100722 11417469

[ejn16099-bib-0009] di Lazzaro, V. , Pilato, F. , Dileone, M. , Profice, P. , Oliviero, A. , Mazzone, P. , Insola, A. , Ranieri, F. , Tonali, P. A. , & Rothwell, J. C. (2008). Low‐frequency repetitive transcranial magnetic stimulation suppresses specific excitatory circuits in the human motor cortex. The Journal of Physiology, 586, 4481–4487. 10.1113/jphysiol.2008.159558 18653655 PMC2614026

[ejn16099-bib-0010] di Lazzaro, V. , Profice, P. , Ranieri, F. , Capone, F. , Dileone, M. , Oliviero, A. , & Pilato, F. (2012). I‐wave origin and modulation. Brain Stimulation, 5, 512–525. 10.1016/j.brs.2011.07.008 21962980

[ejn16099-bib-0011] di Lazzaro, V. , Restuccia, D. , Oliviero, A. , Profice, P. , Ferrara, L. , Insola, A. , Mazzone, P. , Tonali, P. , & Rothwell, J. C. (1998). Effects of voluntary contraction on descending volleys evoked by transcranial stimulation in conscious humans. The Journal of Physiology, 508(Pt 2), 625–633. 10.1111/j.1469-7793.1998.625bq.x 9508823 PMC2230886

[ejn16099-bib-0012] di Lazzaro, V. , Thickbroom, G. W. , Pilato, F. , Profice, P. , Dileone, M. , Mazzone, P. , Insola, A. , Ranieri, F. , Tonali, P. A. , & Rothwell, J. C. (2007). Direct demonstration of the effects of repetitive paired‐pulse transcranial magnetic stimulation at I‐wave periodicity. Clinical Neurophysiology, 118, 1193–1197. 10.1016/j.clinph.2007.02.020 17398148

[ejn16099-bib-0013] Fonov, V. , Evans, A. C. , Botteron, K. , Almli, C. R. , McKinstry, R. C. , & Collins, D. L. (2011). Unbiased average age‐appropriate atlases for pediatric studies. NeuroImage, 54, 313–327. 10.1016/j.neuroimage.2010.07.033 20656036 PMC2962759

[ejn16099-bib-0014] Gentner, R. , Wankerl, K. , Reinsberger, C. , Zeller, D. , & Classen, J. (2008). Depression of human corticospinal excitability induced by magnetic theta‐burst stimulation: Evidence of rapid polarity‐reversing metaplasticity. Cerebral Cortex, 18, 2046–2053. 10.1093/cercor/bhm239 18165282

[ejn16099-bib-0015] Hamada, M. , Galea, J. M. , di Lazzaro, V. , Mazzone, P. , Ziemann, U. , & Rothwell, J. C. (2014). Two distinct interneuron circuits in human motor cortex are linked to different subsets of physiological and behavioral plasticity. The Journal of Neuroscience, 34, 12837–12849. 10.1523/JNEUROSCI.1960-14.2014 25232119 PMC6705319

[ejn16099-bib-0016] Hamada, M. , Hanajima, R. , Terao, Y. , Arai, N. , Furubayashi, T. , Inomata‐Terada, S. , Yugeta, A. , Matsumoto, H. , Shirota, Y. , & Ugawa, Y. (2007). Quadro‐pulse stimulation is more effective than paired‐pulse stimulation for plasticity induction of the human motor cortex. Clinical Neurophysiology, 118, 2672–2682. 10.1016/j.clinph.2007.09.062 17977788

[ejn16099-bib-0017] Huang, Y. Z. , & Rothwell, J. C. (2004). The effect of short‐duration bursts of high‐frequency, low‐intensity transcranial magnetic stimulation on the human motor cortex. Clinical Neurophysiology, 115, 1069–1075. 10.1016/j.clinph.2003.12.026 15066532

[ejn16099-bib-0018] Huang, Y. Z. , Rothwell, J. C. , Edwards, M. J. , & Chen, R. S. (2008). Effect of physiological activity on an NMDA‐dependent form of cortical plasticity in human. Cerebral Cortex, 18, 563–570. 10.1093/cercor/bhm087 17573373

[ejn16099-bib-0019] Iezzi, E. , Conte, A. , Suppa, A. , Agostino, R. , Dinapoli, L. , Scontrini, A. , & Berardelli, A. (2008). Phasic voluntary movements reverse the aftereffects of subsequent theta‐burst stimulation in humans. Journal of Neurophysiology, 100, 2070–2076. 10.1152/jn.90521.2008 18753328

[ejn16099-bib-0020] Kidgell, D. J. , Mason, J. , Frazer, A. , & Pearce, A. J. (2016). I‐wave periodicity transcranial magnetic stimulation (iTMS) on corticospinal excitability. A systematic review of the literature. Neuroscience, 322, 262–272. 10.1016/j.neuroscience.2016.02.041 26917270

[ejn16099-bib-0021] Kotan, S. , Kojima, S. , Miyaguchi, S. , Sugawara, K. , & Onishi, H. (2015). Depression of corticomotor excitability after muscle fatigue induced by electrical stimulation and voluntary contraction. Frontiers in Human Neuroscience, 9, 363. 10.3389/fnhum.2015.00363 26150781 PMC4472998

[ejn16099-bib-0022] Kujirai, K. , Kujirai, T. , Sinkjaer, T. , & Rothwell, J. C. (2006). Associative plasticity in human motor cortex during voluntary muscle contraction. Journal of Neurophysiology, 96, 1337–1346. 10.1152/jn.01140.2005 16723411

[ejn16099-bib-0023] Liao, W. Y. , Sasaki, R. , Semmler, J. G. , & Opie, G. M. (2022). Cerebellar transcranial direct current stimulation disrupts neuroplasticity of intracortical motor circuits. PLoS ONE, 17, e0271311. 10.1371/journal.pone.0271311 35820111 PMC9275832

[ejn16099-bib-0024] Lo, S. , & Andrews, S. (2015). To transform or not to transform: Using generalized linear mixed models to analyse reaction time data. Frontiers in Psychology, 6, 1171. 10.3389/fpsyg.2015.01171 26300841 PMC4528092

[ejn16099-bib-0025] Long, J. , Federico, P. , & Perez, M. A. (2017). A novel cortical target to enhance hand motor output in humans with spinal cord injury. Brain, 140, 1619–1632. 10.1093/brain/awx102 28549131 PMC6059088

[ejn16099-bib-0026] Nudo, R. J. (2013). Recovery after brain injury: Mechanisms and principles. Frontiers in Human Neuroscience, 7, 887. 10.3389/fnhum.2013.00887 24399951 PMC3870954

[ejn16099-bib-0027] Opie, G. M. , Cirillo, J. , & Semmler, J. G. (2018). Age‐related changes in late I‐waves influence motor cortex plasticity induction in older adults. The Journal of Physiology, 596, 2597–2609. 10.1113/JP274641 29667190 PMC6023828

[ejn16099-bib-0028] Opie, G. M. , Sasaki, R. , Hand, B. J. , & Semmler, J. G. (2021). Modulation of motor cortex plasticity by repetitive paired‐pulse TMS at late I‐wave intervals is influenced by intracortical excitability. Brain Sciences, 11, 121. 10.3390/brainsci11010121 33477434 PMC7829868

[ejn16099-bib-0029] Puri, R. , & Hinder, M. R. (2022). Response bias reveals the role of interhemispheric inhibitory networks in movement preparation and execution. Neuropsychologia, 165, 108120. 10.1016/j.neuropsychologia.2021.108120 34915037

[ejn16099-bib-0030] Rossi, S. , Hallett, M. , Rossini, P. M. , & Pascual‐Leone, A. (2009). Safety, ethical considerations, and application guidelines for the use of transcranial magnetic stimulation in clinical practice and research. Clinical Neurophysiology, 120, 2008–2039. 10.1016/j.clinph.2009.08.016 19833552 PMC3260536

[ejn16099-bib-0031] Sale, M. V. , Ridding, M. C. , & Nordstrom, M. A. (2007). Factors influencing the magnitude and reproducibility of corticomotor excitability changes induced by paired associative stimulation. Experimental Brain Research, 181, 615–626. 10.1007/s00221-007-0960-x 17487476

[ejn16099-bib-0032] Sasaki, R. , Hand, B. J. , Semmler, J. G. , & Opie, G. M. (2023). Modulation of I‐wave generating pathways with repetitive paired‐pulse transcranial magnetic stimulation: A transcranial magnetic stimulation‐electroencephalography study. Neuromodulation, 26, 755–766. 36463028 10.1016/j.neurom.2022.10.055

[ejn16099-bib-0033] Sewerin, S. , Taubert, M. , Vollmann, H. , Conde, V. , Villringer, A. , & Ragert, P. (2011). Enhancing the effect of repetitive I‐wave paired‐pulse TMS (iTMS) by adjusting for the individual I‐wave periodicity. BMC Neuroscience, 12, 45. 10.1186/1471-2202-12-45 21592344 PMC3118964

[ejn16099-bib-0034] Stefan, K. , Kunesch, E. , Benecke, R. , Cohen, L. G. , & Classen, J. (2002). Mechanisms of enhancement of human motor cortex excitability induced by interventional paired associative stimulation. The Journal of Physiology, 543, 699–708. 10.1113/jphysiol.2002.023317 12205201 PMC2290505

[ejn16099-bib-0035] Sweatt, J. D. (2016). Neural plasticity and behavior—Sixty years of conceptual advances. Journal of Neurochemistry, 139(Suppl 2), 179–199. 10.1111/jnc.13580 26875778

[ejn16099-bib-0036] Thickbroom, G. W. , Byrnes, M. L. , Edwards, D. J. , & Mastaglia, F. L. (2006). Repetitive paired‐pulse TMS at I‐wave periodicity markedly increases corticospinal excitability: A new technique for modulating synaptic plasticity. Clinical Neurophysiology, 117, 61–66. 10.1016/j.clinph.2005.09.010 16326137

[ejn16099-bib-0003] von Bernhardi, R. , Bernhardi, L. E. , & Eugenín, J. (2017). What is neural plasticity? Advances in Experimental Medicine and Biology, 1015, 1–15. 10.1007/978-3-319-62817-2_1 29080018

[ejn16099-bib-0037] Zhou, Q. , & Poo, M. M. (2004). Reversal and consolidation of activity‐induced synaptic modifications. Trends in Neurosciences, 27, 378–383. 10.1016/j.tins.2004.05.006 15219736

[ejn16099-bib-0038] Ziemann, U. , & Siebner, H. R. (2008). Modifying motor learning through gating and homeostatic metaplasticity. Brain Stimulation, 1, 60–66. 10.1016/j.brs.2007.08.003 20633369

