# Supplementary material for: Effect of current direction and muscle activation on motor cortex neuroplasticity induced by repetitive paired‐pulse transcranial magnetic stimulation
Source: Eur J Neurosci. 2023 Jul 27;58(5):3270–85. doi: 10.1111/ejn.16099 (PMC10946698; doi:10.1111/ejn.16099)
Supplement: Supplementary file 1 — Table S1. Absolute MEP amplitude and SICF data at each time point for all three experiments (mean ± standard deviation). [file EJN-58-3270-s001.docx]

**Supplementary results**

*Experiment 1*: Although RMT did not differ between sessions (*F*_1,60_ = 0.1, *P* = 0.8), stimulation intensities varied between coil orientations (*F*_1,60_ = 98, *P* < 0.05), with *post-hoc* comparisons showing stronger intensities for AP compared to PA (EMD = 12.8 [10.2, 15.4], *P* < 0.05). There was no interaction between factors (*F*_1,60_ = 0.01, *P* = 0.9). Similarly, while AMT did not differ between sessions (*F*_1,44_ = 0.3, *P* = 0.6), stimulation intensities varied between coil orientations (*F*_1,44_ = 91, *P* < 0.05), with *post-hoc* comparisons showing increased intensities for AP stimulation compared to PA (EMD = 12.5 [9.9, 15.1], *P* < 0.05). There was no interaction between factors (*F*_1,44_ = 1.7, *P* = 0.2). Lastly, while TS did not differ between sessions (*F*_1,60_ = 0.46, *P* = 0.5), stimulation intensities varied between coil orientations (*F*_1,60_ = 94, *P* < 0.05), with *post-hoc* comparisons showing increased intensities for AP stimulation compared to PA (EMD = 17.9 [14.2, 21.6], *P* < 0.05). There was no interaction between factors (*F*_1,60_ = 3.3, *P* = 0.07). Stimulation intensities for iTMS varied between sessions (*F*_1,30_ = 68, *P* < 0.05), with comparisons revealing stronger intensities for resting AP compared to resting PA stimulation (EMD = 16.1 [12.1, 20.0], *P* < 0.05).

*Experiment 2*: RMT did not differ between sessions (*F*_1,64_ = 1.4, *P* = 0.2), but stimulation intensities varied between coil orientations (*F*_1,64_ = 250, *P* < 0.05), with *post-hoc* comparisons showing stronger intensities for AP compared to PA (EMD = 14.2 [12.4, 16.0], *P* < 0.05). There was no interaction between factors (*F*_1,64_ = 0.4, *P* = 0.5). Similarly, while AMT did not differ between sessions (*F*_1,64_ = 0.8, *P* = 0.4), stimulation intensities varied between coil orientation (*F*_1,64_ = 110, *P* < 0.05), with *post-hoc* comparisons showing increased intensities for AP stimulation compared to PA (EMD = 13.7 [11.2, 16.3], *P* < 0.05). There was no interaction between factors (*F*_1,64_ = 0.2, *P* = 0.7). While resting TS did not differ between sessions (*F*_1,64_ = 0.6, *P* = 0.4), stimulation intensities varied between coil orientation (*F*_1,64_ = 240, *P* < 0.05), with *post-hoc* comparisons showing increased intensities for AP stimulation compared to PA (EMD = 18.7 [16.3, 21.2], *P* < 0.05). There was no interaction between factors (*F*_1,64_ = 0.1, *P* = 0.8). While active TS also did not differ between sessions (*F*_1,64_ = 0.4, *P* = 0.5), stimulation intensities varied between coil orientation (*F*_1,64_ = 200, *P* < 0.05), with *post-hoc* comparisons showing increased intensities for AP stimulation compared to PA (EMD = 14.9 [12.8, 17.0], *P* < 0.05). There was no interaction between factors (*F*_1,64_ = 2.0, *P* = 0.2). Stimulation intensities for iTMS differed between sessions (*F*_1,32_ = 61, *P* < 0.05), with comparisons revealing stronger intensities for active AP compared to active PA stimulation (EMD = 15.4 [11.4, 19.4], *P* < 0.05).

*Experiment 3*: While RMT did not differ between sessions (*F*_1,50_ = 1.2, *P* = 0.3), stimulation intensities varied between coil orientations (*F*_1,50_ = 210, *P* < 0.05), with *post-hoc* comparisons showing stronger intensities for AP compared to PA (EMD = 16.6 [14.3, 18.9], *P* < 0.05). There was no interaction between factors (*F*_1,50_ = 1.6, *P* = 0.2). Similarly, while TS did not differ between sessions (*F*_1,50_ = 0.3, *P* = 0.6), stimulation intensities varied between coil orientations (*F*_1,50_ = 240, *P* < 0.05), with *post-hoc* comparisons showing increased intensities for AP stimulation compared to PA (EMD = 19.8 [17.2, 22.4], *P* < 0.05). There was no interaction between factors (*F*_1,50_ = 1.0, *P* = 0.03). Stimulation intensities for iTMS varied between sessions (*F*_1,24_ = 10, *P* = 0.004), with comparisons revealing stronger intensities for resting AP compared to active AP stimulation (EMD = 8.2 [2.9, 13.5], *P* = 0.004).

For all Experiments, absolute values for MEP and SICF at each time point are provided in Table S1.

|  |  | iTMS | |  | MEP | | MEP (mV) | | | | | SICF_1.5ms_ | | | | SICF_4.3ms_ | | | |
| --- | --- | --- | --- | --- | --- | --- | --- | --- | --- | --- | --- | --- | --- | --- | --- | --- | --- | --- | --- |
|  |  | Direction | State |  | Direction | State | | Baseline | 5 mins | 30 mins | Baseline | | 5 mins | 30 mins | Baseline | | 5 mins | 30 mins |  |
| Expt 1 (n=16) | |  |  |  |  |  | |  |  |  |  | |  |  |  | |  |  |  |
|  | Session 1 | PA | Rest |  | PA | Rest | | 0.67 ± 0.65 | 0.73 ± 0.66 | 0.60 ± 0.64 | 220.2 ± 152.0 | | 240.5 ± 180.0 | 225.5 ± 177.2 | 113.5 ± 107.2 | | 127.8 ± 147.4 | 119.5 ± 150.8 |  |
|  |  | PA | Rest |  | AP | Rest | | 0.68 ± 0.65 | 0.74 ± 0.75 | 0.70 ± 0.78 | 174.2 ± 108.9 | | 184.2 ± 133.6 | 191.9 ± 152.3 | 119.6 ± 105.0 | | 137.3 ± 127.7 | 133.9 ± 124.3 |  |
|  |  |  |  |  |  |  | |  |  |  |  | |  |  |  | |  |  |  |
|  | Session 2 | AP | Rest |  | PA | Rest | | 0.74 ± 0.69 | 0.85 ± 0.82 | 0.76 ± 0.64 | 193.0 ± 120.1 | | 229.5 ± 165.4 | 257.3 ± 160.9 | 123.7 ± 110.5 | | 139.2 ± 130.9 | 131.3 ± 104.9 |  |
|  |  | AP | Rest |  | AP | Rest | | 0.69 ± 0.62 | 0.94 ± 0.76 | 0.94 ± 0.81 | 192.9 ± 140.9 | | 217.3 ± 144.7 | 216.0 ± 154.3 | 119.6 ± 102.2 | | 180.6 ± 153.3 | 163.4 ± 160.0 |  |
| Expt 2 (n=17) | |  |  |  |  |  | |  |  |  |  | |  |  |  | |  |  |  |
|  | Session 1 | PA | Active |  | PA | Rest | | 1.39 ± 0.81 | 1.59 ± 1.01 | - | 128.0 ± 58.0 | | 147.5 ± 75.7 | - | 103.6 ± 53.0 | | 122.9 ± 70.4 | - |  |
|  |  | PA | Active |  | PA | Active | | 1.76 ± 0.86 | 1.98 ± 1.17 | - | 213.6 ± 126.0 | | 222.1 ± 143.4 | - | 102.1 ± 56.3 | | 119.4 ± 72.0 | - |  |
|  |  | PA | Active |  | AP | Rest | | 1.34 ± 0.89 | 1.54 ± 1.15 | - | 128.6 ± 65.0 | | 143.1 ± 84.6 | - | 97.0 ± 58.4 | | 118.7 ± 73.9 | - |  |
|  |  | PA | Active |  | AP | Active | | 1.62 ± 1.13 | 1.72 ± 1.06 | - | 194.1 ± 105.0 | | 235.1 ± 157.2 | - | 104.2 ± 63.6 | | 112.1 ± 77.4 | - |  |
|  |  |  |  |  |  |  | |  |  |  |  | |  |  |  | |  |  |  |
|  | Session 2 | AP | Active |  | PA | Rest | | 1.25 ± 0.71 | 1.30 ± 0.92 | - | 133.7 ± 75.4 | | 149.9 ± 97.5 | - | 112.1 ± 67.1 | | 116.8 ± 90.5 | - |  |
|  |  | AP | Active |  | PA | Active | | 1.40 ± 0.85 | 1.28 ± 1.03 | - | 244.8 ± 156.3 | | 229.7 ± 158.7 | - | 106.8 ± 89.4 | | 102.8 ± 71.2 | - |  |
|  |  | AP | Active |  | AP | Rest | | 1.19 ± 0.99 | 1.39 ± 1.05 | - | 126.8 ± 91.9 | | 157.9 ± 138.8 | - | 101.4 ± 74.8 | | 126.7 ± 103.8 | - |  |
|  |  | AP | Active |  | AP | Active | | 1.32 ± 0.72 | 1.51 ± 1.09 | - | 221.9 ± 116.6 | | 209.6 ± 134.2 | - | 114.9 ± 58.7 | | 115.3 ± 84.6 | - |  |
| Expt 3 (n=14) | |  |  |  |  |  | |  |  |  |  | |  |  |  | |  |  |  |
|  | Session 1 | AP | Rest |  | PA | Rest | | 0.67 ± 0.55 | 0.59 ± 0.66 | ­- | 244.7 ± 153.4 | | 210.7 ± 136.3 | - | 134.1 ± 96.8 | | 102.9 ± 100.2 | - |  |
|  |  | AP | Rest |  | AP | Rest | | 0.60 ± 0.62 | 0.73 ± 0.91 | ­- | 221.8 ± 150.0 | | 240.9 ± 213.2 | - | 123.3 ± 104.4 | | 137.2 ± 138.9 | - |  |
|  |  |  |  |  |  |  | |  |  |  |  | |  |  |  | |  |  |  |
|  | Session 2 | AP | Active |  | PA | Rest | | 0.80 ± 0.75 | 1.17 ± 1.26 | ­- | 219.4 ± 157.6 | | 290.0 ± 206.9 | - | 141.2 ± 118.4 | | 177.3 ± 144.4 | - |  |
|  |  | AP | Active |  | AP | Rest | | 0.68 ± 0.66 | 0.93 ± 1.03 | ­- | 202.0 ± 168.6 | | 314.0 ± 339.6 | - | 171.5 ± 200.7 | | 226.1 ± 280.0 | - |  |

**Table S1.** Absolute MEP amplitude and SICF data at each time point for all three experiments (mean ± standard deviation).
